# Phyto-Beneficial Traits of Rhizosphere Bacteria: In Vitro Exploration of Plant Growth Promoting and Phytopathogen Biocontrol Ability of Selected Strains Isolated from Harsh Environments

**DOI:** 10.3390/plants11020230

**Published:** 2022-01-17

**Authors:** Gianluigi Giannelli, Franco Bisceglie, Giorgio Pelosi, Beatrice Bonati, Maura Cardarelli, Maria Luisa Antenozio, Francesca Degola, Giovanna Visioli

**Affiliations:** 1 Dipartimento di Scienze Chimiche, della Vita e della Sostenibilità Ambientale, Università di Parma, Parco Area delle Scienze 11/a, 43124 Parma, Italy; gianluigi.giannelli@unipr.it (G.G.); franco.bisceglie@unipr.it (F.B.); giorgio.pelosi@unipr.it (G.P.); beatrice.bonati@unipr.it (B.B.); 2 C.I.R.C.M.S.B.—Consorzio Interuniversitario di Ricerca in Chimica dei Metalli nei Sistemi Biologici, Parma Local Unit, 43124 Parma, Italy; 3 IBPM-CNR, P.le A. Moro 5, 00185 Roma, Italy; maura.cardarelli@uniroma1.it (M.C.); marialuisa.antenozio@uniroma1.it (M.L.A.); 4 Dipartimento di Biologia e Biotecnologie, Università Sapienza di Roma, 00185 Roma, Italy

**Keywords:** antifungal metabolites, biocontrol agents, plant growth promoting rhizobacteria, phytopathogen antagonists, siderophore production, stressful soils

## Abstract

Beneficial interactions between plants and some bacterial species have been long recognized, as they proved to exert various growth-promoting and health-protective activities on economically relevant crops. In this study, the growth promoting and antifungal activity of six bacterial strains, *Paenarthrobacter ureafaciens*, *Beijerinckia fluminensis*, *Pseudomonas protegens*, *Arthrobacter* sp., *Arthrobacter defluii*, and *Arthrobacter nicotinovorans*, were investigated. The tested strains resulted positive for some plant growth promoting (PGP) traits, such as indole-3-acetic acid (IAA), 1-aminocyclopropane-1-carboxylate-deaminase (ACC-deaminase), siderophore production, and solubilization of phosphates. The effect of the selected bacteria on *Arabidopsis thaliana* seedlings growth was assessed using different morphological parameters. Bacterial activity against the phytopathogenic fungal species *Aspergillus flavus*, *Fusarium proliferatum*, and *Fusarium verticillioides* was also assessed, since these cause major yield losses in cereal crops and are well-known mycotoxin producers. Strains Pvr_9 (*B. fluminensis*) and PHA_1 (*P. protegens*) showed an important growth-promoting effect on *A. thaliana* coupled with a high antifungal activity on all the three fungal species. The analysis of bacterial broths through ultra performance liquid chromatography–mass spectrometry (UPLC–MS) and liquid chromatography–electrospray ionization–mass spectrometry (LC–ESI–MS/MS) confirmed the presence of potential PGP-compounds, among these are desferrioxamine B, aminochelin, asperchrome B, quinolobactin siderophores, and salicylic acid.

## 1. Introduction

The rhizosphere is a complex ecosystem in which many relationships are established between bacteria, fungi, and plant root apparatus, and represents the main source of nutrients for plant growth [1]. In particular, many soil microbes have established good relationships with plants, supporting their growth and health, for example helping plants to manage both biotic and abiotic stress [2,3,4]. In particular, plant growth promoting rhizobacteria (PGPR) are microorganisms, which form symbiotic interactions with plant roots, promoting plant health and productivity through different mechanisms such as production of plant hormones (auxins, cytokinin, and gibberellins); inhibition of plant senescence; N_2_ fixation; phosphate solubilization and mineralization of other nutrients; and siderophores production [5]. In addition, being present in the rhizosphere, PGPR may also be endophytic (PGPE) (for example, by colonizing the plant’s tissues), symbiotic (for example, by colonizing the interior of the roots of specific plants by forming nodules), or phyllospheric (i.e., they can be found on the surfaces of plant leaves and stems) [6].

The majority of the most known PGPR belong to the genera *Alcaligenes*, *Arthrobacter*, *Azospirillum*, *Azotobacter*, *Bacillus*, *Burkholderia*, *Enterobacter*, *Klebsiella*, *Pseudomonas*, *Rhizobium*, and *Serratia* [7]. PGPR beneficial effects on plants include an increase in root growth and shoot biomass, chlorophyll content, nutrient uptake, total protein content, hydraulic activity, abiotic stress tolerance, shoot and root weights, and a delayed senescence. PGPR are, thus, often employed as biofertilizers [8].

Besides being determinant for plant health and soil fertility, the interactions between beneficial microbes and plant rhizosphere can also exert direct, positive effects against phytopathies. PGPR can suppress diseases by directly synthesizing pathogen-antagonizing compounds, as well as by triggering plant immune responses [9]. Some PGPR have been found to possess several chemotypical traits that make them potential antifungal agents for biocontrol purposes. They can produce siderophores, antimicrobials, lytic enzymes, and various extracellular metabolites which can interfere with, if not completely inhibit, the growth of different, devastating phytopathogenic fungal species with a broad host range [10]. For example, *Pseudomonas* spp. strains isolated from the rhizosphere of alfalfa and clover plants growing on extremely poor pseudogley soil showed interesting antifungal activity against *Trichoderma viride*, *Aspergillus fumigatus*, and *Aspergillus niger* [11], while plant-promoting *Pseudomonas fluorescens* and *Bacillus* spp. strains from a PGPR collection were found to effectively inhibit three spore-forming genera (*Alternaria* spp., *Fusarium* spp., *Bipolaris* spp.) [12]. Again, *Phytophthora capsici*, a cucumber pathogen, was successfully suppressed by specific isolates of *Pseudomonas stutzeri* and *B. amyloliquefaciens* [13]. Recently, a battery of bacteria isolated from the rhizosphere of crops cultivated in different agroecosystems of Pakistan was screened for their biocontrol potential against a range of fungal phytopathogens, showing antagonistic activity against *Fusarium oxysporum*, *F. moniliforme*, *Rhizoctonia solani*, *Colletotrichum gloeosporioides*, *C. falcatum*, *Aspergillus niger*, and *A. flavus* [14]; the antimicrobial effect, which was ascribed to the individuation of antifungal metabolites such as specific antibiotics and cell wall degrading enzymes, was accompanied by the production of a number of compounds recognized as plant growth promoters (hormones and siderophores), suggesting that these PGPR can be exploited for dual-purpose strategies based on the application of a single formulation acting as biopesticide and biofertilizer [15]. It is worthy of consideration that specific bacterial siderophores have been demonstrated to possess direct antifungal activity (often affecting spore germination) against phytopathogens such as *F. oxysporum*, *F. udum*, *A. niger*, *A. flavus*, and *Sclerotium rolfsii* [16,17,18]; pyoverdine and pyocheline in particular, produced by *P. aeruginosa* and *Burkholderia* spp., have been attributed the most relevant antifungal activities of these bacterial species [19].

Interestingly, other molecules produced by some rhizosphere bacteria and also involved in the plant disease resistance show antifungal properties, as it is the case of salicylic acid (SA) and its derivatives [20,21,22,23,24].

In this panorama, the aim of this work was (i) to characterize bacterial isolates—derived from different soil and rhizosphere environments—for their capacity to improve *Arabidopsis thaliana* growth, (ii) to test their potential activity as biocontrol agents against phytopathogenic fungi species, and (iii) to identify possible molecules involved in plant mineral nutrition or with antimicrobial activity.

## 2. Results

### 2.1. Evaluation of the Bacterial Strains Properties

In this study, a deeper characterization of six different bacterial strains previously isolated from different environments was performed. As reported in Table 1, the selected strains Pvr_5, and Pvr_9, Bioch_2, Bioch_7, NCr-1 showed some features of PGPR as high in vitro IAA production and 1-aminocyclopropane-1-carboxylic acid (ACC) deaminase activity (Table 1) [25,26,27], as well as the production of siderophores, which was diagnosed by using a qualitative method [25,26,27]. In this work, a liquid chrome azurol S (CAS) assay was used to evaluate the siderophore production in a semiquantitative manner. To this purpose, the bacteria selected were cultured in two different media: succinate minimal salt (SMS) medium, containing a low amount of iron, and succinic medium (SM), completely deprived of iron. The CAS assay revealed that all bacterial strains are able to produce, in both growth media, molecules involved in chelating ferric ions, as indicated by the elevated percent siderophore units (PSU) values observed (Table 1).

The capacities to solubilize phosphate and bacterial protease activity were also tested with qualitative test assays (Figure S1); PHA_1 [28] represents the only strain able to solubilize phosphate while Pvr_5, NCr-1, Bioch_2 and Bioch_7 showed protease activity. Microbial peptidases play a central role in the nitrogen cycle in the soils as they make nitrogen available for plants [29]. Finally, Bioch_7 and Pvr_9 showed an in vitro capacity to form biofilm.

### 2.2. Effect of Bacterial Inoculation on A. thaliana Morphological Features

**Figure 1:**
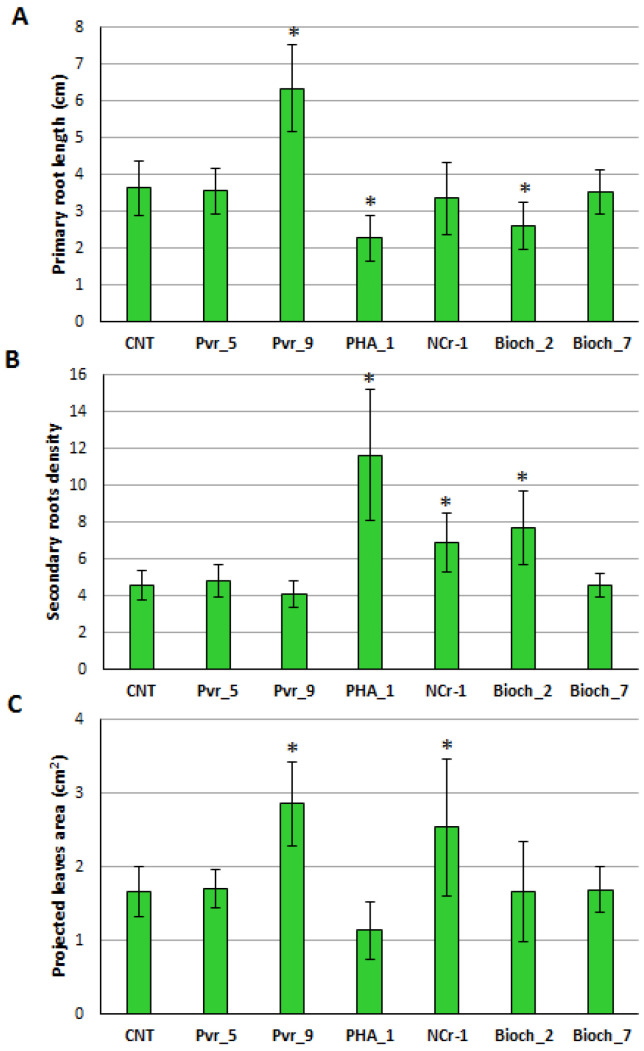
Effect of bacterial inoculation on *Arabidopsis* 14-day-old seedling morphological traits. (**A**) Primary root length, expressed in cm; (**B**) secondary roots density, expressed as number of secondary roots per cm of primary root; (**C**) projected leaves area, expressed in square cm. Data presented are means of 30 biological replicates ± standard deviation (S.D.). Asterisks indicate statistically significant differences between control condition (not inoculated) and treatments (inoculated), according to ANOVA and Tukey’s test (*p* < 0.05).

The primary root length measurement was carried out on 14-day-old *A. thaliana* seedlings after single bacterial strains inoculation on seeds. As reported in Figure 1A, Pvr_9 proved to be the only strain able to significantly increase (*p* < 0.05) the primary root length of seedlings. On the contrary, plantlets grown in the presence of PHA_1 and Bioch_2 showed a slight although significant reduction (*p* < 0.05) of the primary root length with respect to the control condition, while no significant differences were observed for plants grown in association with Pvr_5, NCr-1, and Bioch_7 (Figure S2).

The density of the seedlings secondary roots treated with bacteria was also evaluated: in PHA_1-inoculated plantlets the value was about three times significantly higher (*p* < 0.05) than in the control condition, and a significant increase (*p* < 0.05) was also observed for NCr-1 and Bioch_2-treated seedlings. No significant differences were detected in the case of the application of the other strains (Figure 1B).

The last parameter measured was the leaf projection area: seeds inoculation with Pvr_9 and NCr-1 were found to be the only condition that determined a significant increase (*p* < 0.05) of values in treated plantlets (Figure 1C).

### 2.3. Antifungal Activity against Selected Phytopathogenic Fungi

**Figure 2:**
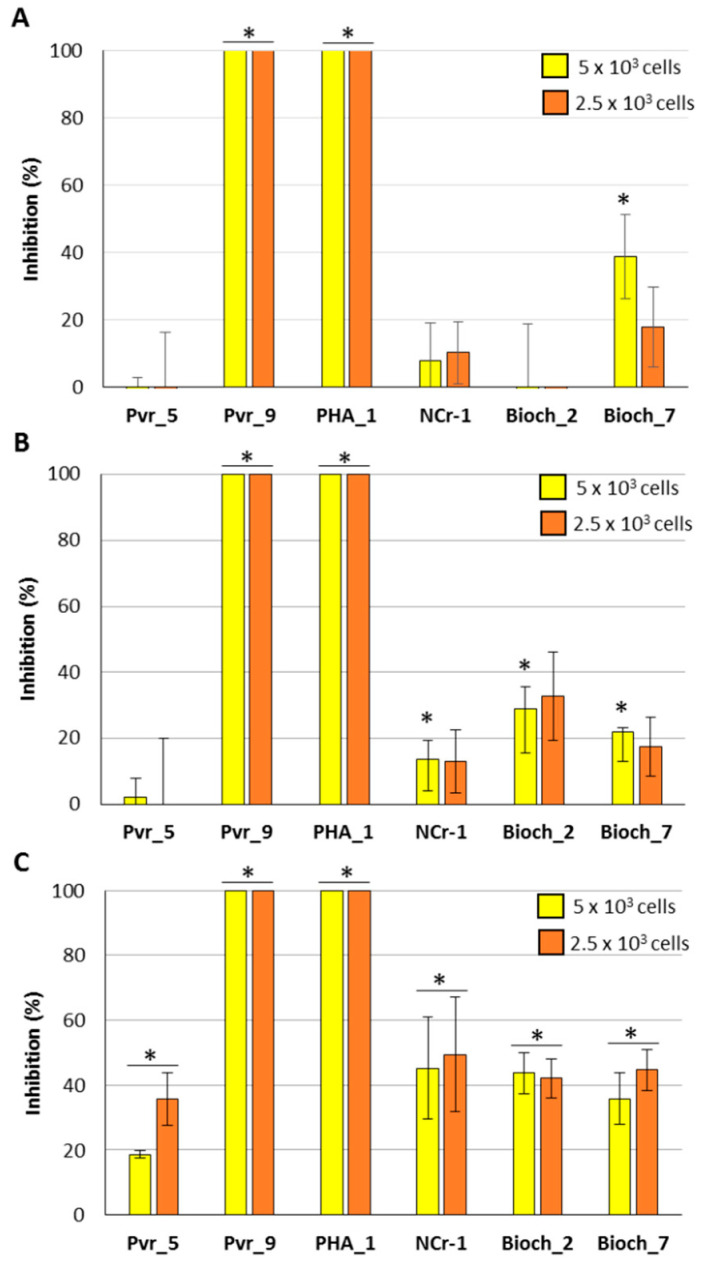
Antifungal activity of bacteria co-inoculum against *A. flavus* (**A**), *F. verticillioides* (**B**), and *F. proliferatum* (**C**). Concentrations of 5 or 2.5 × 10^3^ bacterial cells were co-inoculated with 5 × 10^3^ of fungal conidia. Data are presented as percentage inhibition with respect to the control (only fungal cultures) and are the means of six biological replicates ± standard deviation (S.D.). Asterisks indicate statistically significant differences between control and co-inoculated cultures according to ANOVA and Tukey’s test (*p* < 0.05).

**Figure 3:**
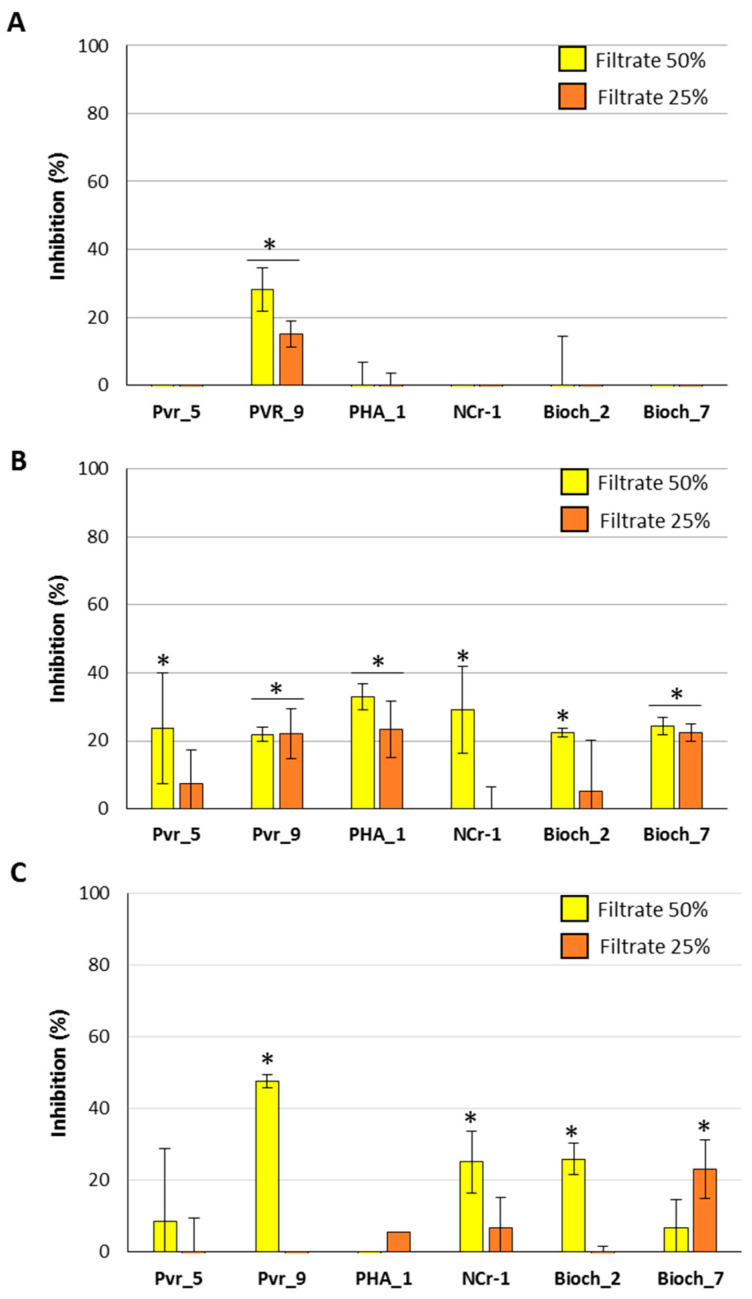
Antifungal activity of filtered bacterial culture broth against *A. flavus* (**A**), *F. verticillioides* (**B**), and *F. proliferatum* (**C**). Cultures of 5 × 10^3^ of fungal conidia/well were amended with 25 or 50% bacterial broth. Data are presented as percentage inhibition with respect to the control (fungal cultures only) and are the means of six biological replicates ± standard deviation (S.D.). Asterisks indicate statistically significant differences between control and co-inoculated cultures according to ANOVA and Tukey’s test (*p* < 0.05).

Bacterial strains were tested for their direct activity against the phytopathogenic species *A. flavus*, *F. verticillioides*, and *F. proliferatum*: the antifungal potential was assayed by both co-inoculating bacteria cells and fungal conidia and cultivating fungal strains in presence of bacterial filtrated culture medium (Figure 2 and Figure 3). Bacteria were tested at two different concentrations (2.5 and 5 × 10^3^ cells/well). The highest antifungal activity was obtained against *A. flavus* with the co-inoculation, at both the bacterial concentration of Pvr_9 and PHA_1, which reached 100% inhibition (Figure 2A). The same effectiveness was observed against the other two fungal species investigated, *F. verticillioides* and *F. proliferatum* (Figure 2B,C). Co-inoculation with Bioch_7 determined approximately a 40% inhibition in *A. flavus* and 20% in *F. verticillioides* growth at the highest cell concentration (5 × 10^3^), while *F. proliferatum* was subjected to a 40% inhibition at both the concentrations used (Figure 2).

When inoculating 5 × 10^3^ cells of NCr-1 and Bioch_2, a 20% and 35% reduction of growth in *F. verticillioides* was observed, respectively; an inhibition at both the concentrations used against *F. proliferatum* was also detected (Figure 2B,C). On the contrary, no antifungal activity against *A. flavus* was found (Figure 2A). Bacterial strain Pvr_5 was able to impair the growth of *F. proliferatum* but not of the other two fungal species (Figure 2).

The antifungal effect of the bacterial broths was assayed by adding 25 and 50% (*v*/*v*) to the fungal culture medium, where 5 × 10^3^ conidia/well was inoculated. Through the administration of the filtered bacterial broth, only Pvr_9 was shown able to interfere with the growth of *A. flavus* (Figure 3A).

The inhibition was about 30 and 15% by amending the medium with 50 and 25% of filtered culture broth, respectively. Pvr_9 broth was also effective in reducing the mycelium growth of the other two fungal species: 20% inhibition was observed against *F. verticilliodes* at both broth concentrations used while around 50% inhibition was obtained when 50% of broth was added to *F. proliferatum* (Figure 3B,C). Administration of 50 and 25% of PHA_1 broth determined 35 and 20% inhibition on *F. verticillioides*, respectively (Figure 3B); on the contrary, no effects were achieved on *F. proliferatum*, which instead resulted in inhibition (50%) by NCr-1 and Bioch_2 filtrates (Figure 3C). Interestingly, the same fungal species resulted in more effect by 25 than 50% of Bioch_7 broth, a peculiarity that might be attributed to a combined effect of specific and nonspecific inhibitors that differentially act on mycelium development (Figure 3C).

Finally, 20% inhibition of *F. verticilliodes* growth was recorded when using Bioch_7 filtered culture broth at every percentage, and 50% of Pvr_5, NCr-1, and Bioch_2.

### 2.4. Identification of Potentially Beneficial Molecules for Plant in Bacterial Broths

UPLC–MS and LC–ESI–MS analyses of SMS and SM culture broths from bacteria were conducted in order to identify compounds possibly linked to the plant-promoting and/or fungal-inhibitory activities observed. Salicylic acid was found in the culture broth of Pvr_5 and Bioch_7 grown in SMS medium, and in Pvr_9, PHA_1, and NCr-1 grown in SMS and SM media. Among the molecules identified, UPLC–MS analysis revealed the presence of the hydroxamate desferrioxamine B in the SMS broth of Pvr_5, while LC–ESI–MS/MS analysis was able to detect the presence of the catecholate aminochelin in the SM broth of Pvr_9. In SM medium, NCr-1 was found to produce the hydroxamate siderophore asperchrome B, and Bioch_2 the carboxylate quinolobactin (Table 2).

## 3. Discussion

The beneficial interaction between plants and some rhizobacteria has been long recognized, as they proved to exert various growth-promoting and health-protective activities on economically relevant crops. However, although many of them express similar PGPR activity, some typically possess more than one beneficial trait, facilitating in different ways the interfacing of their plant symbionts with the environment. Thus, since the nature and the mechanism of such positive biological interactions have still to be completely clarified, and because PGPR species from same genus often exhibit different interactions with the phytosphere [30,31], the exploration and characterization of new, potentially beneficial strains is highly desirable as well. With this purpose, the bacterial strains analyzed in this study were chosen amongst a previously described panel, containing isolates recovered from the rhizosphere—or the surrounding soil—of plants grown in stressful environments; the mining of harsh ecosystems is in fact considered particularly promising for seeking plant-beneficial bacteria, having the microbiota from these areas subjected to evolutionary pressures that have, in turn, led to adaptive features related to a more effective stress response of their hosts than plants (and the relevant rhizosphere) found in cultivated land [32]. Recently, the screening of rhizobacteria associated with halophytes and drought-tolerant plants inhabiting salty and arid areas of the Mediterranean basin successfully allowed for individualizing isolates that showed interesting abiotic stress-contrasting and biocontrol traits [33], validating the exploration of similar, extreme environments as a rewarding strategy for the individuation of PGP strains.

Identified at the genera and species level by 16S rDNA sequencing, and only partially characterized for their putative PGPR properties, bacteria strains elected for this work belonged to differently demanding environments: Pvr_5 and Pvr_9 were isolated from the rhizosphere of the As-hyperaccumulating fern *Pteris vittata* [27], PHA_1 from a soil rich in hydrocarbons [28], NCr-1 was found to be an endophyte of the Ni-hyperaccumulator *Noccaea caerulescens* [25], and Bioch_2 and Bioch_7 were isolated from a third-year biochar-amended soil [26]. In this work, for this purpose, a deeper characterization of the selected bacterial strains was performed. In particular, a study on their plant-protective/promoting characteristics and potentials was carried out, performing observations of the direct effects on the growth parameters of the model plant species *A. thaliana* and on selected phytopathogenic fungi.

Among the tested strains, Pvr_9 was considered the most interesting, due to the important effects shown as both plant growth promoter and biocontrol agent against some phytopathogenic fungi. The molecular characterization previously conducted showed a homology with the bacterial species *Beijerinckia fluminensis* [27], belonging to a genus that is still poorly characterized for its putative PGPR properties.

On the contrary, strain PHA_1, which shows a significant increase in *Arabidopsis* secondary root formation and interesting features as a biocontrol agent against phytopathogenic fungi tested, belong to the well-known *Pseudomonas* genus, which group includes various interesting species that show microbial biocontrol features and PGP traits, and that has proven to be very versatile, with great potential from an agronomic point of view. Many works described *P. protegens* as an effective antimicrobial agent. Cesa-Luna and collaborators [34] evaluated the ability of *P. protegens* strain EMM-1 against different fungal species, reporting significant activity against *Aspergillus* spp. and *Fusarium* spp. *P. protegens* strain AS15 was shown to be an effective biocontrol agent against *A. flavus*, whose growth and aflatoxin production were lowered on rice grains after the bacterial co-inoculation [35]. The powerful antifungal activity of this species was confirmed by our results; in fact, PHA_1 proved to be highly inhibitory on the fungal growth, especially when the conidia were forced to germinate in the presence of the bacterial cells in co-inoculation assays; in fact, the inhibition reached 100%, independent of the bacterial cell concentration. In addition to the production of antimicrobial compounds, which have also been suggested by the presence, in the genome of the strain FD6, of 12 putative gene clusters for secondary metabolites production, including the antibiotics 2,4-diacetylphloroglucinol (2,4-DAPG), pyoluteorin (PLT), and pyrrolnitrin (PRN) [21], various PGP traits were also reported for some *P. protegens* strains, as the production of siderophores, ammonia, and IAA, the phosphate solubilization [36]. Here, the evaluation of the association of PHA_1 with *A. thaliana* showed a significant increase in the number of secondary roots per cm of primary root, in accordance with what has been recently observed on maize roots inoculated with *Pseudomonas* PS01 strain [37].

Bioch_2, Bioch_7, and NCr-1 belong to the *Arthrobacter* genus and Pvr_5 to the *Paenarthrobacter* genus, in which many plant endophytes are grouped. The plant growth promoting traits of the genus *Arthrobacter* is well documented; their capabilities to produce auxins, siderophores, and ACC deaminase, as well as to exert a P-solubilizing activity, are widely reported, and often associated with a reduction of plant stress when *Arthrobacter* is inoculated. Safdarian et al. [38] showed that *A. nitroguajacolicus* was able to act as a plant growth promoting bacterium on maize under salt stress condition; Tchuisseu Tchakounté et al. [39] recovered, from the maize rhizosphere, 29 isolates belonging to the *Arthrobacter* genus and showed that many possessed at least one of the tested PGP traits. The presence of PGP trait within the *Arthrobacter* genus was also confirmed by Kumar et al. [40]. In his work *A. chlorophenolicus* showed NH_3_ production, HCN production, N_2_ fixation, IAA production, and P-solubilizing capabilities.

All the bacteria tested were siderophore producers and, with the exclusion of Pvr_5, all the strains were more or less able to interfere with the mycelium growth of *Fusarium*. As previously reported, siderophores can mitigate the toxic effect of fusaric acid produced by the genus *Fusarium* on *Pseudomonas protegens* Pf-5 [41].

In addition, all the bacterial strains selected showed high siderophore activity. There is increasing interest on siderophore-producing bacteria and siderophore molecules, not only for their possible role in iron bioavailability for plant nutrition, but also to their suppressive activity against fungal phytopathogens. Jin et al. showed that IAA and soil microbial siderophores are both important for Fe uptake by plants [42]. The siderophore pyoverdine produced by *P. fluorescens* was shown to have an important role in the iron uptake of *A. thaliana* [43]. Masalha et al. [44] showed the importance of microbial activity for the iron acquisition in *Zea mays* and in *Helianthus annuus*. Siderophores produced by *Pseudomonas syringae* are biologically active against *Fusarium oxysporum* and other plant pathogenic fungi, through suppression of sporulation and of fungal growth [45].

For this purpose, as an objective of this study, the identification of the siderophores produced by bacterial strains could help to better investigate possible molecules involved not only in plant nutrition, but also in bacterial antimicrobial activity against the phytopathogenic fungi tested. Among the molecules with hydroxamate functional group, asperchrome B and desferrioxamine B are well-known siderophores, which are produced by various species of bacteria and fungi. Desferrioxamine B in particular is a linear trilhydroxamic acid siderophore [46]. In addition to chelating Fe (III), desferrioxamine B is also able to bind, for instance, Cu (II), Se (II), Pb (II), Co (III), Mn (III), and Bi (III) [47]. Desferrioxamine B and its chemical derivatives have received much attention because of their particular biological activity. The applications in the medical field of this molecule concerns its use in antimalarial prophylaxis, in a strategy based on the use of antibiotics linked to siderophores to facilitate their entry into cells (Trojan horse strategy), its use as a fluorescent sensor, and in treatment in cases of patients suffering from metal poisoning and iron overload [48].

Among the molecules with catecholate functional groups, we find aminochelin produced by Pvr_9. A characterization of the chemical properties of aminochelin was carried out by [49]. Aminochelin is a triprotic acid with two chatecol protons and one amine proton, with a simple bidentate structure and a high hydrophobicity. This structure enables Fe (III) chelation and to solubilize ferric hydroxides. The carboxylate quinolobactin, an 8-hydroxy-4-methoxy-2-quinoline carboxylic acid, was identified as a siderophore for *Pseudomonas fluorescens* ATCC 17400 [50].

Finally, the carboxylate containing salicylic acid (SA) was found to be produced by most of the bacterial strains tested. In addition to its use by bacteria to maintain iron-limiting growth conditions [20], SA production was reported to also exert an inhibitory potential against several postharvest pathogens, including *Botrytis cinerea* [21], *F. oxysporum* [22], *Penicillium expansum* [23], and *Rhizopus stolonifer* [24].

## 4. Materials and Methods

### 4.1. Microorganisms Used in This Study and Growth Conditions

Six bacterial strains isolated from different sources were selected for this work from a collection of PGPR present in our laboratory: Bioch_2 (homologous to *Arthrobacter defluvii*) and Bioch_7 (homologous to *Arthrobacter nicotinovorans*) strains were previously isolated by a maize-derived biochar utilized as amendment in a three year poplar short rotation coppice plantation [26]; Ncr-1 (homologous to *Arthrobacter* sp.) is an endophyte strain isolated from the roots of the Ni-hyperaccumulator *Noccaea caerulescens* [27]; PHA_1 (homologous to *Pseudomonas protegens*) was isolated from a soil contaminated with hydrocarbons [28]; Pvr_5 (homologous to *Paenarthrobacter ureafaciens*); and Pvr_9 (homologous to *Beijerinckia fluminensis*) were isolated from the rhizosphere of the As-hyperaccumulator *Pteris vittata* fern [25]. PGPR characteristics were reported in Table 1.

The aflatoxigenic *A. flavus* strain CR10 and two strains of *F. verticilloides* and *F. proliferatum* were used to assess the antifungal activity of bacteria. All the fungal strains were maintained on potato dextrose agar medium (PDA; Oxoid, Thermo Fisher Scientific Waltham, MA, USA). For conidia production, *A. flavus* was cultured on PDA for 14 days at 28 °C, while *Fusarium* strains were cultured on nutrient synthetic medium (SNA; KH_2_PO_4_ 1.0 g L^−1^, KNO_3_ 1.0 g L^−1^, MgSO_4_∙7H_2_O 0.5 g L^−1^, KCl 0.5 g L^−1^ Glucose 0.2 g L^−1^, Sucrose 0.2 g L^−1^, Agar 15.0 g L^−1^) for 20 days.

### 4.2. Assessment of PGP Traits of Bacterial Strains

Inorganic phosphate solubilization activity of the selected bacteria was assessed using Pikovskaya (PVK) medium (dextrose 10 g L^−1^, yeast extract 0.5 g L^−1^, calcium phosphate 5 g L^−1^, ammonium sulfate 0.5 g L^−1^, potassium chloride 0.2 g L^−1^, magnesium sulphate 0.1 g L^−1^, manganese sulfate 0.0001 g L^−1^, ferrous sulfate 0.0001 g L^−1^, agar 10 g L^−1^) [51]. Bacterial strains were streaked on PVK agar medium and incubated for 5 days at 28 °C. The phosphate solubilization was assessed by the visualization of a clear halo around the bacterial colony.

Protease activity was evaluated in skim milk agar plate medium (casein hydrolysate 10 g L^−1^, yeast extract 5 g L^−1^, NaCl 4 g L^−1^, skim milk powder 20 g L^−1^, agar 10 g L^−1^). Bacterial strains were streaked and incubated for 5 days at 28 °C. Protease production was determined by the presence of a clear halo surrounding the bacterial colony [52].

Biofilm formation was assessed following the protocol described by O’Toole, with some modifications [53]. An overnight bacterial culture in plate count agar (PCA) medium was diluted 1:100 in fresh PCA liquid medium and 100 μL was inoculated in a well of a 96-well plate and then placed in static growth for 5 days at 28 °C. After incubation, the medium was discarded and the plate submerged in water two times. Then, 125 μL of a 0.1% solution of crystal violet for each well was added and the plate incubated for 15 min at room temperature. The plate was rinsed 3 times with water and, after water removal, dried for 2 h. A volume of 125 μL of 30% acetic acid solution was added; after 15 min of incubation, absorbance was quantified at 595 nm wavelength.

To measure siderophore activity, bacteria were grown in either SMS (sucrose 1% (*w*/*v*), (NH_4_)_2_SO_4_ 0.1%, K_2_HPO_4_ 0.2%, MgSO_4_ 0.05%, NaCl 0.01%, yeast extract 0.05%, CaCO_3_ 0.05%, tryptophan 0.5 mg mL^−1^) or SM (succinic acid 4%, (NH_4_)_2_SO_4_ 1%, KH_2_PO_4_ 3%, K_2_HPO_4_ 0.1%, MgSO_4_ 0.2%) for three days; cultures were then centrifuged to remove the cells and 500 μL of supernatant was added to the same volume of CAS solution, then incubated for 20 min at RT. The CAS assay solution contained 6 mL of 10 mM hexadecyltrimethylammonium bromide (HDTMA), 1.5 mL of 1 mM FeCl_3_, 7.5 mL of 2 mM CAS, 4.307 g of piperazine, and 6.25 mL of 12 M HCl, then diluted to 100 mL with double-distilled water according to Jeong et al. 2014 [54]. To quantify the activity of siderophores produced by each strain, absorbance at 630 nm was determined, and the result was expressed as siderophore unit (percentage) [55]. Three replicates per bacterial colony were analyzed. The results are expressed as mean ± S.D.

### 4.3. Seed Bacterial Inoculation and Plants Growth Parameters

*Arabidopsis thaliana* (L.) Heynh. Columbia-0 seeds were used. Seeds were surface sterilized for 5 min with 40% NaClO solution, then washed four times with double-distilled sterile water. After washing, seeds were kept three days in the dark at 4 °C to allow the synchronization of germination. Bacterial strains were grown in 3 mL of Luria and Bertani medium on shaking (130 rpm) at 28 °C for 24 h. Seed inoculation with the different strains was performed as follows: seeds were kept for 1.5 h in a bacterial solution (1 × 10^8^ cells mL^−1^) on shaking, then recovered and plated on half strength MS [56] + 1% *w*/*v* sucrose agar medium. Plates were incubated in a vertical position in an environmentally controlled chamber growth (24 °C; 16/8 h light/dark photoperiod; 120 μmol m^−2^ s^−1^ photosynthetically active radiation, 75% relative humidity (RH)) for germination and root elongation. Plantlets were collected after 14 days for growth measurements. Primary root length, rosette area, and number of lateral roots were measured on 14-day-old plants inoculated or not with bacteria isolates. The number of total lateral roots was normalized for the total length of the primary root. All the measures were performed using ImageJ software (available at http://rsb.info.nih.gov/ij/ accessed on 20 September 2021; developed by Wayne Rasband, National Institutes of Health, Bethesda, MD, USA). The results are expressed as mean ± S.D. A total of 30 plants per treatment were analyzed.

### 4.4. Direct Antifungal Activity Assay

Antifungal activity of bacteria was assessed through a 96-multiwell plate cultivation system. In the first assay, bacteria were grown for three days in PCA (enzymatic digest of casein 10.0 g L^−1^, yeast extract 2.5 g L^−1^, dextrose 1.0 g L^−1^) liquid medium on shaking at 28 °C, then aliquots of cells were recovered and washed twice in bidistilled water; bacterial cells were then properly diluted and co-inoculated in 96-multiwell plates, in a final volume of 200 μL of PCA liquid medium, with fungal conidia suspensions at the same concentration. Plates were incubated in the dark in static growth at 28 °C.

A second assay was performed to assess the antifungal activity of bacteria broth: bacteria were grown for three days in PCA liquid medium on shaking at 28 °C; cultures were then centrifuged at 4000 rpm for 20 min and the cells discarded. Each broth was filtered with a 0.22 μm filter. Then, spores of each fungal species (5 × 10^3^) were inoculated in 96-multiwell plates with 50 or 100 μL of filtered broth to a final volume of 200 μL/well of PCA medium, corresponding to the 25 and 50% (*v*/*v*) of the culture, respectively.

In both assays, biomass production was assessed after ten days of incubation for *A. flavus*, while *F. verticilloides* and *F. proliferatum* were evaluated after 14 days; mycelia from single wells were recovered, slightly dried on paper, and weighted. Values were expressed as percentage of inhibition with respect to the control. Inocula were performed in quadruplicate, and experiments were performed in triplicate.

### 4.5. Identification of Potential Plant Growth Beneficial Molecules by Bacterial Strains

Bacterial broths obtained from a three-day culture were centrifuged and the supernatant was recovered and added with methanol at a 3:1 volume ratio. Then, four volumes of ethanol were added and the samples were left undisturbed overnight at 4 °C [44]. The supernatant was recovered and concentrated at 45 °C with a vacuum rotary evaporator and utilized for the following analyses.

#### 4.5.1. Detection of Functional Groups

Each sample was subjected to two different tests for the detection of the iron-chelating functional groups. The tetrazolium test was employed to verify the presence of hydroxamate type of siderophore [57]. Briefly, a pinch of tetrazolium salt was added in a test tube to which 1–2 drops of 2 N NaOH was added and subsequently 1 mL of test sample. Immediate development of a deep red color was taken as a positive reaction by hydroxamate-type siderophore. Moreover, Arnow’s test was used to determine functional groups belonging to the catecholate type of siderophores [58]. This method is based on the reaction between catechol and nitrite–molybdate reagent, in acidic conditions, producing a yellow color. The color changes to an intense orange-red in alkaline conditions. For this purpose, 1.0 mL of culture filtrate was combined with 1.0 mL of HCl 0.5 mol∙L^−1^. Subsequently, 1.0 mL of nitrite–molybdate reagent was added and then 1.0 mL of NaOH 1.0 mol∙L^−1^. The assay was incubated at room temperature for approximately 5 min to allow full color development. As a blank control sample, 1.0 mL of deionized water was used. Nitrite–molybdate reagent was prepared by dissolving 10 g of sodium nitrite and 10 g of sodium molybdate in 100 mL of deionized water. The presence of an orange-red color solution detects the catecholate type siderophore. The color intensity depends on the amount of catechol present [58,59].

#### 4.5.2. UPLC Determination

To better identify the siderophore, the solutions were also tested by means of ultra-performance liquid chromatography (UPLC) (Waters S.p.A. Sesto San Giovanni (MI), Italy) associated with electrospray ionization mass spectrometry (Waters Acquity UPLC/ESI–MS, single quadrupoles detector) (Waters S.p.A. Sesto San Giovanni (MI), Italy). To separate active components, each sample was injected and separated on a C18 column (Waters Acquity UPLC BEH300 C18 1.7 µm, 2.1∙50 mm) using a gradient of 0.1% aqueous formic acid (A) and acetonitrile (B) as mobile phase (0–5 min 1.5–45% B, 5–16 min 45–100% B and then 16–19 min 100% B; flow rate 0.2 mL∙min^−1^; temperature 30 °C). The capillary and cone voltages in ESI mode were 3.8 kV and 25 V, respectively [59,60]. Ion transfer capillary was heated at 300 °C. Cone and desolvation gas flow was, respectively, at 100 and 480 L∙h^−1^. Positive-ion full-scan mass spectra were recorded from *m*/*z* 50 to 2000.

#### 4.5.3. LC–ESI–MS/MS Determination

High resolution mass spectrometry was performed on the samples using a HPLC DIONEX Ultimate3000 interfaced with a LTQ-Orbitrap XL Thermo Fisher Scientific (Waltham, MA, USA). Samples were injected on an Aeris Peptide 3.6u XB-C18 2.1 mm × 15 cm (Phenomenex; Via M. Serenari, 15/D, 40013 Castel Maggiore (BO), Italy). The mobile phase consisted of water with 0.1% formic acid (solvent A) and methanol with 0.1% formic acid (solvent B); gradient: 0–5 min 99% A, 5–35 min from 99% A to 5% A, 35–40 min 5% A, 40–41 min from 5% A to 99% A, 41–50 min 99% A; flow rate was 0.2 mL/min; column temperature 35 °C; injection volume 5 μL. Samples were acquired in positive and negative mode. Electrospray ionization at positive (spray voltage 3 kV; capillary voltage 13 V; source temperature 275 °C; tube lens 100 V; sheath gas flow rate 40; aux gas flow rate 10; and sweep gas flow rate 5) and negative (spray voltage 3.2 kV; capillary voltage −35 V; source temperature 275 °C; tube lens −110 V; sheath gas flow rate 40; aux gas flow rate 10; and sweep gas flow rate 5) ion modes. The mass data acquisition was performed by four scan events. Data were analyzed using a database dedicated to microbial siderophores and created by Prof. Samuel Bertrand (http://bertrandsamuel.free.fr/siderophore_base/index.php released on 8 June 2011, accessed on 14 December 2021); compounds were identified through the main adduct encountered using LC–ESI–MS, namely, [M + H]^+^, [M − 2H + Fe]^+^, and [M − H]^−^.

### 4.6. Statistical Analyses

For statistical analyses, one-way analysis of variance (ANOVA) was used in the Past 4.06b software [61]. Results of plant growth measures and antifungal activity were analysed by Tukey’s test; differences were considered significant at *p* < 0.05.

## 5. Conclusions

Amongst the bacterial strains evaluated, Pvr_9 was found to possess the best characteristics for both promoting the plant growth and acting as biocontrol agent against phytopathogens. The preliminary results achieved not only confirmed the mining of harsh environments as a promising tool for the individuation of potential PGPR, but also provide important clues about the direct antagonistic effect of these strains on *Aspergillus* and *Fusarium* species relevant to crops. Future investigations devoted to deepening and clarifying the mechanism ruling the positive effects on the growth of plants—and in particular of economically important crops—are needed before any possible application in agricultural systems can be proposed. In particular, more research is desirable to elucidate the direct antimicrobial potential of the siderophores identified, which would support the possible use of such bacteria as biocompetitors able to act against phytopathogenic fungal species in different synergistic ways.

## Figures and Tables

**Table 1:** Characteristics of PGPR bacteria strains isolated from different soil types, rhizosphere, and endosphere samples.

Strains	Homology	Siderophore Production (PSU) ^(a)^		IAA Production (mg L^−1^)	ACC Deaminase Activity ^(b)^	Phosphate Solubilization Ability ^(c)^	Protease Activity ^(d)^	Biofilm Formation (Abs Units)	References
		SMS	Succinic						
Pvr_5	*Paenarthrobacter ureafaciens* (98.16%)	88.64 ± 0.74	91.5 ± 1.05	62.48 ± 6.3	+	-	+	0.037 ± 0.010	[25]
Prv_9	*Beijerinckia fluminensis* (100%)	91.90 ± 0.11	70.7 ± 2.60	82.08 ± 1.7	+	-	-	1.048 ± 0.141	[25]
Bioch_2	*Arthrobacter defluii* (98%)	91.29 ± 0.56	85.91 ± 4.70	44.02 ± 2.3	+	-	+	0.1 ± 0.007	[26]
Bioch_7	*Arthrobacter nicotinovorans* (99%)	92.33 ± 0.70	89.02 ± 1.12	58.65 ± 4.2	+	-	+	0.216 ± 0.032	[26]
NCr-1	*Arthrobacter* sp. (99%)	93.04 ± 0.08	58.78 ± 2.78	25.6 ± 1.3	+	-	+	0.059 ± 0.003	[27]
PHA_1	*Pseudomonas protegens* (98%)	90.38 ± 0.09	76.89 ± 4.94	n.d.	n.d.	+	-	0.134 ± 0.007	[28]

^(a)^ Siderophore production on SMS and succinic media (see Material and Methods for media composition). ^(b)^ ACC deaminase activity: (-) no bacterial growth on medium containing 1-aminocyclopropane-1-carboxylate as the only N source; (+) bacterial growth on medium containing 1-aminocyclopropane-1-carboxylate as the only N source. ^(c)^ Phosphate solubilization: (-) absence of solubilization halo; (+) presence of solubilization halo. ^(d)^ Protease activity. (-) absence of solubilization halo; (+) presence of solubilization halo. Data are average of three independent experiments ± S.D.

**Table 2:** Identified molecules produced by bacteria and their relative functional groups, along with the growth medium and the technique used for the analysis (n.d., not detected).

Isolates	Functional Group	SMS Medium	Succinic Medium
Pvr_5	Carboxylate	Salicylic Acid (UPLC–MS)	n.d.
	Hydroxamate	Desferrioxamine B (UPLC–MS)	n.d.
Pvr_9	Carboxylate	Salicylic Acid (UPLC–MS; LC–ESI–MS/MS)	Salicylic Acid (UPLC–MS; LC–ESI–MS/MS)
	Catecholate	n.d.	Aminochelin (LC–ESI–MS/MS)
PHA_1	Carboxylate	Salicylic Acid (UPLC–MS; LC–ESI–MS/MS)	Salicylic Acid (UPLC–MS; LC–ESI–MS/MS)
NCr-1	Carboxylate	Salicylic Acid (UPLC–MS; LC–ESI–MS/MS)	Salicylic Acid (UPLC–MS; LC–ESI–MS/MS)
	Hydroxamate	n.d.	Asperchrome B (UPLC–MS)
Bioch_2	Carboxylate	Quinolobactin (UPLC–MS)	n.d.
Bioch_7	Carboxylate	Salicylic Acid (UPLC–MS)	n.d.

## Data Availability

Data is contained within the article and Supplementary Material.

## Supplementary Materials

The following supporting information can be downloaded at: https://www.mdpi.com/article/10.3390/plants11020230/s1, Figure S1: Phosphate solubilization assay; Figure S2: Primary root elongation.
