# Supplementary material for: Phyto-Beneficial Traits of Rhizosphere Bacteria: In Vitro Exploration of Plant Growth Promoting and Phytopathogen Biocontrol Ability of Selected Strains Isolated from Harsh Environments
Source: Plants (Basel). 2022 Jan 17;11(2):230. doi: 10.3390/plants11020230 (PMC8779669; doi:10.3390/plants11020230)
Supplement: Supplementary file 1 [file plants-11-00230-s001.zip › plants-1543154-supplementary.pdf]

Supplementary Materials

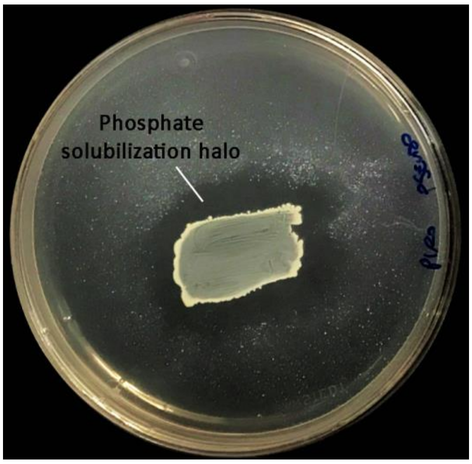

Figure S1. Phosphate solubilization assay. Example of PHA_1 positive solubilization halo surrounding the bacterial colony.

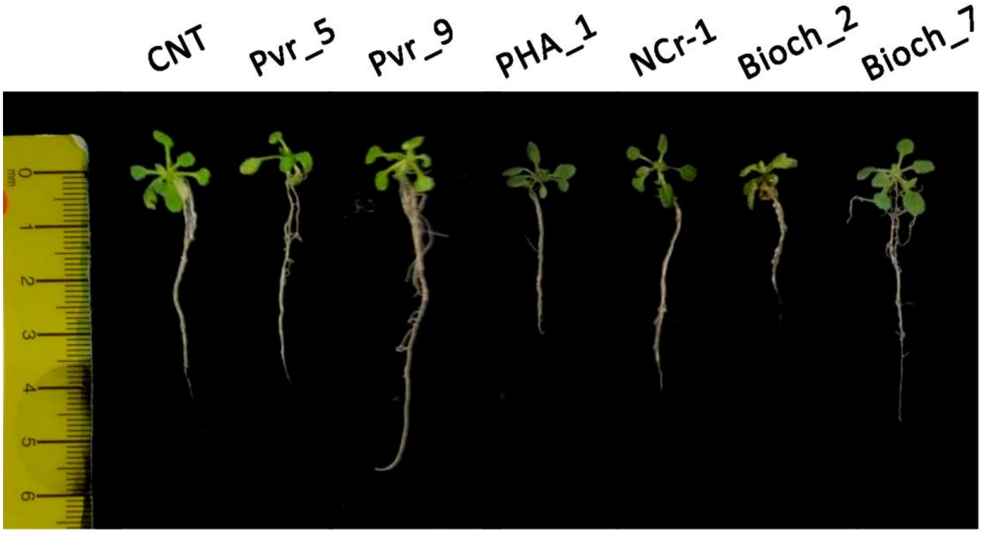

Figure S2. Primary root elongation. Examples of *A. thaliana* 14-days-old seedlings after single bacterial strains inoculation on seeds.
